# Supplementary material for: Diverse Cretaceous larvae reveal the evolutionary and behavioural history of antlions and lacewings
Source: Nat Commun. 2018 Aug 22;9:3257. doi: 10.1038/s41467-018-05484-y (PMC6105666; doi:10.1038/s41467-018-05484-y)
Supplement: Supplementary file 1 — Supplementary Information [file 41467_2018_5484_MOESM1_ESM.pdf]

**Supplementary Information**

# Diverse Cretaceous larvae reveal the evolutionary and behavioural history of antlions and lacewings

**Badano et al.**

**Supplementary Note 1**

List of synapomorphies supporting the main clades of Myrmeleontiformia obtained under implied weighting (k=9.21875). The strict consensus cladogram with inferred unambiguous character changes is shown in the Supplementary Figure 1. Clade names follows ref. 1.

*Geoneuroptera (Myrmeleontiformia + Ithonidae)*[1]. Non homoplasious apomorphies: 9:1, gular region with a small sclerite limited to anterior portion of head; 37:0, specialized terminal seta on flagellum absent.

*Total group Myrmeleontiformia.* Non homoplasious apomorphies: 10:1, hypostomal bridge present; 12:2, posterior tentorial grooves on anterior third of head capsule; 42:0, maxillary base slightly retracted, maxillary groove short, one third as long as ventral head capsule. Homoplasious apomorphies: 4:0, temple region present; 20:1, setae on anterior margin of clypeolabrum present; 58:0, neck region present.

*Stem-Myrmeleontiformia (*Macleodiella + Cladoger*).* Homoplasious apomorphies: 46:1, mandibular teeth present; 51:1, pseudoteeth present.

*Crown-group Myrmeleontiformia.* Non homoplasious apomorphies: 3:1, dolichaster-type setae present; 30:1, prominent antennal tubercle present; 54:1, length of labial palpus at most 1/3 of mandible length. Homoplasious apomorphies: 5:1 posterior prominence of temple quadrate.

*Psychopsidae, inclusive of* Acanthopsychops *and* Aphthartopsychops. Non homoplasious apomorphies: 18:1, Chaetotaxy of the dorsal side of the head capsule inserted on papillae; 21:2, setae on the anterior margin of clypeolabrum stout. Homoplasious apomorphies: 23:1, rostrum present.

*Myrmeleontoidea*[1]. Non homoplasious apomorphies: 1:1, body shape ovoid; 32:2, length of antenna at most 1/3 of mandible length; 35:2, diamater of flagellomeres less than half scape diameter; 56:1, premental element (palpiger) strongly developed and ventrally flattened. Homoplasious apomorphies: 62:1, head foramen, dorsal; 77:0, empodium absent.

*Nymphidae.* Non homoplasious apomorphies: 48:0, one mandibular tooth. Homoplasious apomorphies: 34:0, antennal pedicel extremely short, as long as one flagellomere; 46:1, mandibular

teeth present; 59:1, neck region short; 65:1, one mesothoracic setiferous process; 68:1, one metathoracic setiferous process.

*Nymphinae, inclusive of* Nymphavus. Homoplasious apomorphies: 24:1, spiniform processes on anterior margin of clypeolabrum present; 118: 1, camouflaging behaviour present.

*Nemopteridae + (total group Myrmeleontidae + Ascalaphidae)*. Non homoplasious apomorphies: 21:0, type of setae on the anterior margin of clypeolabrum: dolichasters; 57:1, sensory pit of apical palpomere present; 60:1, median dorsal cervical apodeme present. *Nemopteridae*. Non homoplasious apomorphies: 19:1, clypeolabrum narrow, covering half of anterior margin of head between mandibles. Homoplasious apomorphies: 30:0, antennal tubercle absent.

*Total group Myrmeleontidae + Ascalaphidae*. Homoplasious apomorphies: 35:1, flagellomeres smaller than scape diameter; 65:2, two mesothoracic setiferous processes; 68:2, two metathoracic setiferous processes.

*Total group Myrmeleontidae + Ascalaphidae, exclusive of* Electrocaptivus. Homoplasious apomorphies: 5:0, posterior prominence of temples smooth; 26:1, ocular tubercle present; 46:1, mandibular teeth present; 59:1, neck region short.

*Stem-Myrmeleontidae + Ascalaphidae,* Adelpholeon + *(*Diodontognathus + Mesoptynx). Non homoplasious apomorphies: 48:1, two mandibular teeth.

*Crown-group Myrmeleontidae + Ascalaphidae.* Non homoplasious apomorphies: 7:1, dorsal depression of the head capsule deep; 75:1, tarsus and tibia of metathoracic leg fused. Homoplasious apomorphies: 100:1, abdominal segment 8 with odontoid processes.

*Ascalaphidae*. Non homoplasious apomorphies: 102:1, odontoid processes with setae. Homoplasious apomorphies: 5:1, posterior prominence of temples quadrate.

*Myrmeleontidae, including* Pristinofossor. Non homoplasious apomorphies: 74:1, metathoracic legs more robust than mesothoracic legs. Homoplasious apomorphies: 78:1, Metathoracic leg, pretarsal claws enlarged.

*Crown Myrmeleontidae*. Non homoplasious apomorphies: 106:1, abdominal sternite 9 with specialized digging setae on ventral surface. Homoplasious apomorphies: 105:1, abdominal segment 9 wider than long; 109:1, rastra prominent and sclerotized.

| Species | Specimen code | BL (mm) | HL (mm) | HW (mm) | ML (mm) | HW /HL | ML/ HL |
|---|---|---|---|---|---|---|---|
| *Macleodiella electrina* | AMNH JCZ-Bu30 | 3.1 | 0.8 | 0.4 | 0.8 | 0.5 | 1 |
| *Cladofer huangi* | NIGP152466 | 4.9 | 0.8 | 1.09 | 1.93 | 1.4 | 2.4 |
| *Acanthopsychops triaina* | AMNH JCZ-Bu404 | - | 1.43 | 1.18 | 1.55 | 0.8 | 1.0 |
| *Aphthartopsychops scutatus* | AMNH JCZ-Bu197 | 1.03 | 0.41 | 0.48 | 0.39 | 1.2 | 0.9 |
| | BA12015 | 2.87 | 0.67 | 0.87 | 1.24 | 1.3 | 1.9 |
| | AMNH JCZ-Bu33A | 1.45 | 0.39 | 0.50 | 0.90 | 1.3 | 2.0 |
| *Nymphavus progenitor* | NIGP164047 | 4.10 | 1.20 | 1.68 | 1.93 | 1.4 | 1.6 |
| | NIGP164051 | 3.74 | 0.86 | 1.20 | 1.43 | 1.4 | 1.7 |
| | NIGP164058 | 5.93 | 0.96 | 1.46 | 1.73 | 1.5 | 1.8 |
| *Electrocaptivus xui* | BA1701 | 4.92 | 1.34 | 1.49 | 1.25 | 1.1 | 0.9 |
| *Burmitus tubulifer* | BA12013 | 2.67 | 0.73 | 0.89 | 1.10 | 1.2 | 1.5 |
| *Adelpholeon lithophorus* | AMNH JCZ-Bu31 | 4.56 | 1.52 | 1.3 | 1.82 | 0.8 | 1.2 |
| | BA12011 | 5.02 | 1.89 | 1.96 | 2.50 | 1.0 | 1.3 |
| | BA12012 | 4.41 | 1.30 | 1.30 | 1.67 | 1.0 | 1.3 |
| | BA12017 | 2.32 | 0.62 | 0.66 | 0.79 | 1.1 | 1.3 |
| | NIGP164050 | 2.98 | 0.95 | 0.97 | 1.21 | 1.0 | 1.3 |
| | BA12014 | 1.61 | 0.63 | 0.72 | 0.81 | 1.1 | 1.3 |
| *Diodontognathus papillatus* | NIGP164059 | 6.03 | 1.22 | 1.21 | 1.63 | 1.0 | 1.3 |
| | NIGP164044 | 2.50 | 0.78 | 0.79 | 1.05 | 1.0 | 1.3 |
| | NIGP164046 | 5.30 | 1.40 | 1.30 | 1.98 | 0.9 | 1.4 |
| | NIGP164048 | 2.00 | 0.62 | 0.62 | 0.87 | 1.0 | 1.4 |
| | NIGP164049 | 3.06 | 1.36 | 1.47 | 1.79 | 1.1 | 1.3 |
| | NIGP164060 | 4.27 | 1.37 | 1.38 | 1.91 | 1.0 | 1.4 |
| | AMNH JCZ-Bu29 | - | 0.60 | 0.61 | 0.81 | 1.0 | 1.3 |
| *Mesoptynx unguiculatus* | NIGP164043 | 3.83 | 1.21 | 0.96 | 1.50 | 0.8 | 1.2 |
| *Pristinofossor rictus* | AMNH JCZ-Bu304 | 2.88 | 0.61 | 0.65 | 1.39 | 1.0 | 2.2 |

**Supplementary Table 1. Measurements of examined specimens.** Body length (BL) was measured from head (excluding jaws) to tip of abdomen; head length (HL) was measured ventrally from distal to proximal margin, head width (HW) was taken just below eye tubercles; mandible length (ML) was measured from apex to base.

| Traits 74-114<br># run | Dependent<br>LML run | Independent<br>LML run | LogBF |
|---|---|---|---|
| 1 | -43.521016 | -45.444684 | 3.847336 |
| 2 | -44.202049 | -45.639137 | 2.874176 |
| 3 | -44.225336 | -45.677979 | 2.905286 |
| 4 | -44.285509 | -45.701205 | 2.831392 |
| 5 | -44.3025 | -45.716509 | 2.828018 |
| 6 | -44.311106 | -45.905787 | 3.189362 |
| 7 | -44.386034 | -45.909364 | 3.04666 |
| 8 | -44.391026 | -46.007091 | 3.23213 |
| 9 | -44.467471 | -46.037641 | 3.14034 |
| 10 | -44.489277 | -46.045431 | 3.112308 |
| 11 | -44.509175 | -46.04905 | 3.07975 |
| 12 | -44.663942 | -46.087099 | 2.846314 |
| 13 | -44.709479 | -46.104079 | 2.7892 |
| 14 | -44.785693 | -46.158827 | 2.746268 |
| 15 | -44.822555 | -46.23636 | 2.82761 |
| 16 | -44.850431 | -46.287056 | 2.87325 |
| 17 | -44.886763 | -46.485093 | 3.19666 |
| 18 | -44.930996 | -46.486826 | 3.11166 |
| 19 | -45.28959 | -46.692849 | 2.806518 |
| 20 | -45.395301 | -46.754621 | 2.71864 |
| **Average** | -44.57126245 | -46.0713344 | **3.0001439** |

Interpretation: **Correlated**

**Supplementary Table 2.** Summary of 20 independent correlation analyses using traits 74 (metathoracic leg) and 114 (digging behaviour). LML: Log Marginal Likelihood. LogBF: Log Bayes Factors.

| **Traits 78-114 # run** | **Dependent LML run** | **Independent LML run** | **LogBF** |
|---|---|---|---|
| 1 | -45.631444 | -50.96479 | 10.666692 |
| 2 | -45.855279 | -51.098438 | 10.486318 |
| 3 | -45.905877 | -51.176394 | 10.541034 |
| 4 | -45.996604 | -51.192495 | 10.391782 |
| 5 | -46.0189 | -51.207239 | 10.376678 |
| 6 | -46.141089 | -51.227795 | 10.173412 |
| 7 | -46.264613 | -51.34966 | 10.170094 |
| 8 | -46.467704 | -51.355558 | 9.775708 |
| 9 | -46.486538 | -51.357194 | 9.741312 |
| 10 | -46.639069 | -51.381202 | 9.484266 |
| 11 | -46.690834 | -51.596146 | 9.810624 |
| 12 | -46.74739 | -51.652966 | 9.811152 |
| 13 | -46.889442 | -51.666654 | 9.554424 |
| 14 | -46.910239 | -51.709835 | 9.599192 |
| 15 | -46.963255 | -51.730432 | 9.534354 |
| 16 | -47.025091 | -51.737775 | 9.425368 |
| 17 | -47.165689 | -51.800864 | 9.27035 |
| 18 | -47.219167 | -51.814311 | 9.190288 |
| 19 | -47.405071 | -51.865549 | 8.920956 |
| 20 | -47.495451 | -52.236684 | 9.482466 |
| **Average** | -46.5959373 | -51.50609905 | **9.8203235** |

Interpretation: **Strongly correlated**

**Supplementary Table 3.** Summary of 20 independent correlation analyses using traits 78 (pretarsal claws) and 114 (digging behaviour). LML: Log Marginal Likelihood. LogBF: Log Bayes Factors.

| Traits 93-118<br># run | Dependent<br>LML run | Independent<br>LML run | LogBF |
|---|---|---|---|
| 1 | -38.029285 | -45.079333 | 14.100096 |
| 2 | -38.037895 | -45.566795 | 15.0578 |
| 3 | -38.0539 | -45.604986 | 15.102172 |
| 4 | -38.100645 | -45.755218 | 15.309146 |
| 5 | -38.212651 | -45.961872 | 15.498442 |
| 6 | -38.219522 | -45.983044 | 15.527044 |
| 7 | -38.242751 | -46.129702 | 15.773902 |
| 8 | -38.329322 | -46.130722 | 15.6028 |
| 9 | -38.371485 | -46.588379 | 16.433788 |
| 10 | -38.388221 | -47.635948 | 18.495454 |
| 11 | -38.447843 | -47.774 | 18.652314 |
| 12 | -38.481274 | -49.366286 | 21.770024 |
| 13 | -38.507984 | -49.424466 | 21.832964 |
| 14 | -38.569226 | -49.476367 | 21.814282 |
| 15 | -38.589683 | -49.545658 | 21.91195 |
| 16 | -38.650676 | -50.203924 | 23.106496 |
| 17 | -38.978303 | -50.329325 | 22.702044 |
| 18 | -39.004271 | -50.409331 | 22.81012 |
| 19 | -39.132799 | -50.618929 | 22.97226 |
| 20 | -39.136496 | -50.699212 | 23.125432 |
| **Average** | -38.4742116 | -47.91417485 | **18.8799265** |

Interpretation: **Very strongly correlated**

**Supplementary Table 4.** Summary of 20 independent correlation analyses using traits 93 (abdominal segments 1-7, type of dorsal setiferous processes) and 118 (camouflaging behaviour). LML: Log Marginal Likelihood. LogBF: Log Bayes Factors.

| Traits 94-118<br>**# run** | Depended<br>LML run | Independent<br>LML run | LogBF |
|---|---|---|---|
| 1 | -40.312428 | -44.505595 | 8.386334 |
| 2 | -40.511625 | -44.520683 | 8.018116 |
| 3 | -40.55217 | -44.8187 | 8.53306 |
| 4 | -40.572504 | -44.855137 | 8.565266 |
| 5 | -40.672319 | -44.917178 | 8.489718 |
| 6 | -40.680417 | -45.09268 | 8.824526 |
| 7 | -40.682231 | -45.47256 | 9.580658 |
| 8 | -40.790267 | -45.622101 | 9.663668 |
| 9 | -40.882294 | -45.649712 | 9.534836 |
| 10 | -41.054715 | -45.773229 | 9.437028 |
| 11 | -43.80116 | -46.672981 | 5.743642 |
| 12 | -43.872123 | -47.22299 | 6.701734 |
| 13 | -44.102048 | -48.678953 | 9.15381 |
| 14 | -44.283729 | -49.672045 | 10.776632 |
| 15 | -44.292706 | -49.843987 | 11.102562 |
| 16 | -44.312462 | -49.984377 | 11.34383 |
| 17 | -44.320533 | -50.100619 | 11.560172 |
| 18 | -44.368425 | -50.193337 | 11.649824 |
| 19 | -44.36919 | -50.287417 | 11.836454 |
| 20 | -44.47105 | -50.305768 | 11.669436 |
| **Average** | -42.4452198 | -47.20950245 | **9.5285653** |

Interpretation: **Strongly correlated**

**Supplementary Table 5.** Summary of 20 independent correlation analyses using traits 94 (first abdominal segments 1-2, type of ventral setiferous processes) and 118 (camouflaging behaviour). LML: Log marginal likelihood. LogBF: Log Bayes Factors.

| **Traits 108-114 # run** | **Dependent LML run** | **Independent LML run** | **LogBF** |
|---|---|---|---|
| 1 | -52.154028 | -45.672005 | -12.964046 |
| 2 | -53.298322 | -45.888415 | -14.819814 |
| 3 | -53.377989 | -45.921316 | -14.913346 |
| 4 | -53.389538 | -45.977005 | -14.825066 |
| 5 | -53.491906 | -46.028667 | -14.926478 |
| 6 | -53.619942 | -46.173979 | -14.891926 |
| 7 | -53.63137 | -46.211617 | -14.839506 |
| 8 | -53.661359 | -46.237277 | -14.848164 |
| 9 | -53.794989 | -46.323949 | -14.94208 |
| 10 | -53.973044 | -46.331054 | -15.28398 |
| 11 | -54.038405 | -46.376238 | -15.324334 |
| 12 | -54.287556 | -46.408872 | -15.757368 |
| 13 | -54.351696 | -46.535047 | -15.633298 |
| 14 | -54.419254 | -46.606282 | -15.625944 |
| 15 | -54.720598 | -46.730748 | -15.9797 |
| 16 | -54.790288 | -46.763948 | -16.05268 |
| 17 | -54.96612 | -46.981351 | -15.969538 |
| 18 | -55.105909 | -47.086434 | -16.03895 |
| 19 | -56.287544 | -47.134054 | -18.30698 |
| 20 | -57.70819 | -47.610492 | -20.195396 |
| **Average** | -54.25340235 | -46.4499375 | **-15.6069297** |

Interpretation: **Weakly correlated**

**Supplementary Table 6.** Summary of 20 independent correlation analyses using traits 108 (rastra) and 114 (digging behaviour). LML: Log marginal likelihood. LogBF: Log Bayes Factors.

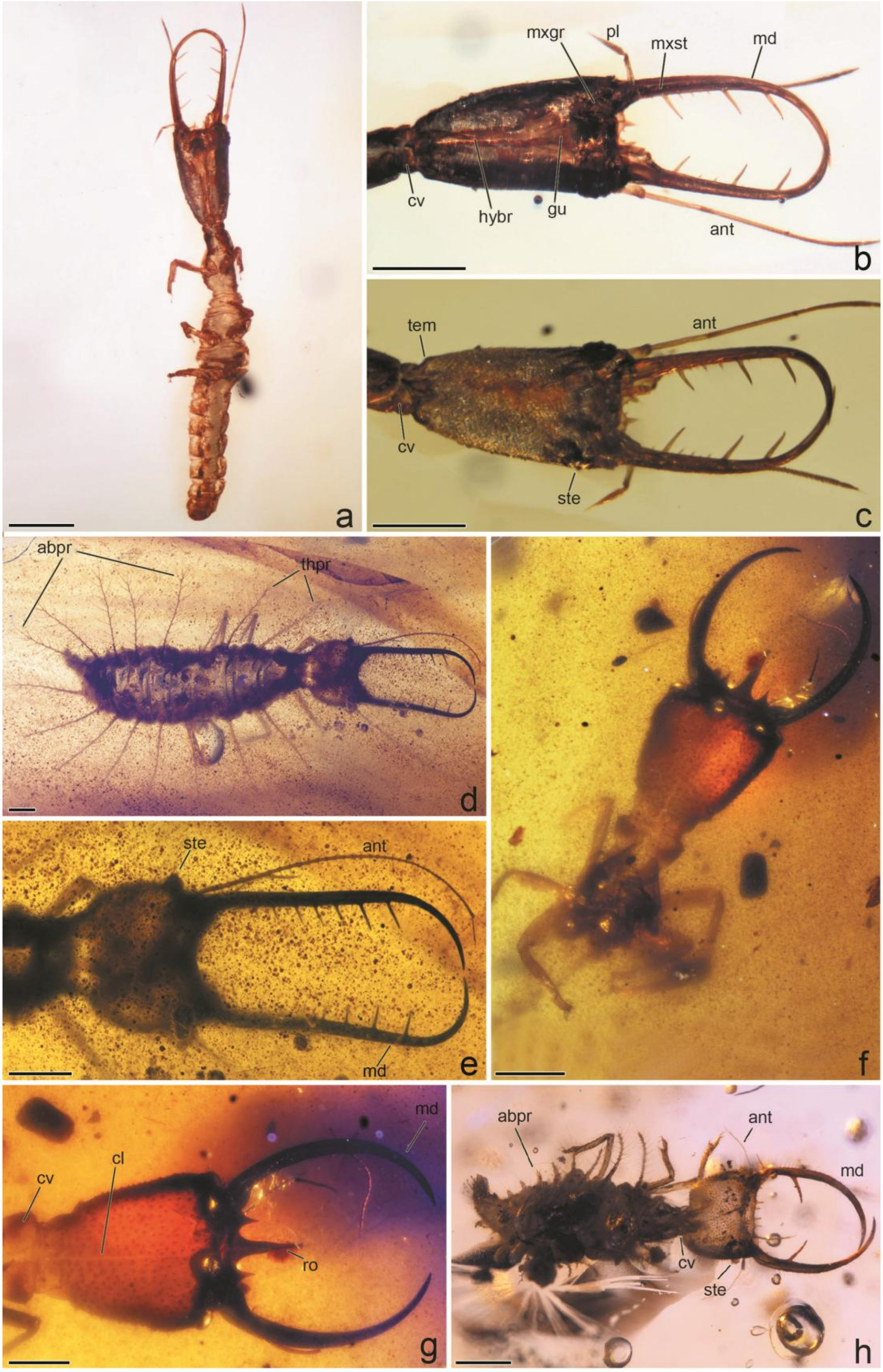


**Supplementary Figure 1.** Diversity of the larvae of Myrmeleontiformia in mid-Cretaceous Burmese amber. **a**, **b**, **c** *Macleodiella electrina* gen. et sp. nov., holotype AMNH JCZ-Bu30: **a** ventral view; **b** head, ventral view; **c** head, dorsal view. **d**, **e** *Cladofer huangi* gen. et sp. nov., holotype, NIGP152466: **d** habitus; **e** head, dorsal view. **f**, **g** *Acanthopsichops triaina* gen. et sp. nov., holotype AMNH JCZ-Bu404: **f** head, dorsal view; **g** habitus, dorsal view. **h** *Pristinofossor rictus* gen. et sp. nov., holotype AMNH JCZ-Bu304, detail of mandible. Abbreviations: abpr, abdominal processes; ant, antenna; cl, cleavage line, cv, cervix; gu, gular sclerite; hs, head sclerite; hybr, hypostomal bridge; md, mandible; mxgr, maxillary groove; mxst, maxillary stylet; pl, labial palpus; ro, rostrum; sp, spiracle; ste, stemmata; tem, temple; thpr, thoracic processes. Scale bars: 0.5 mm.

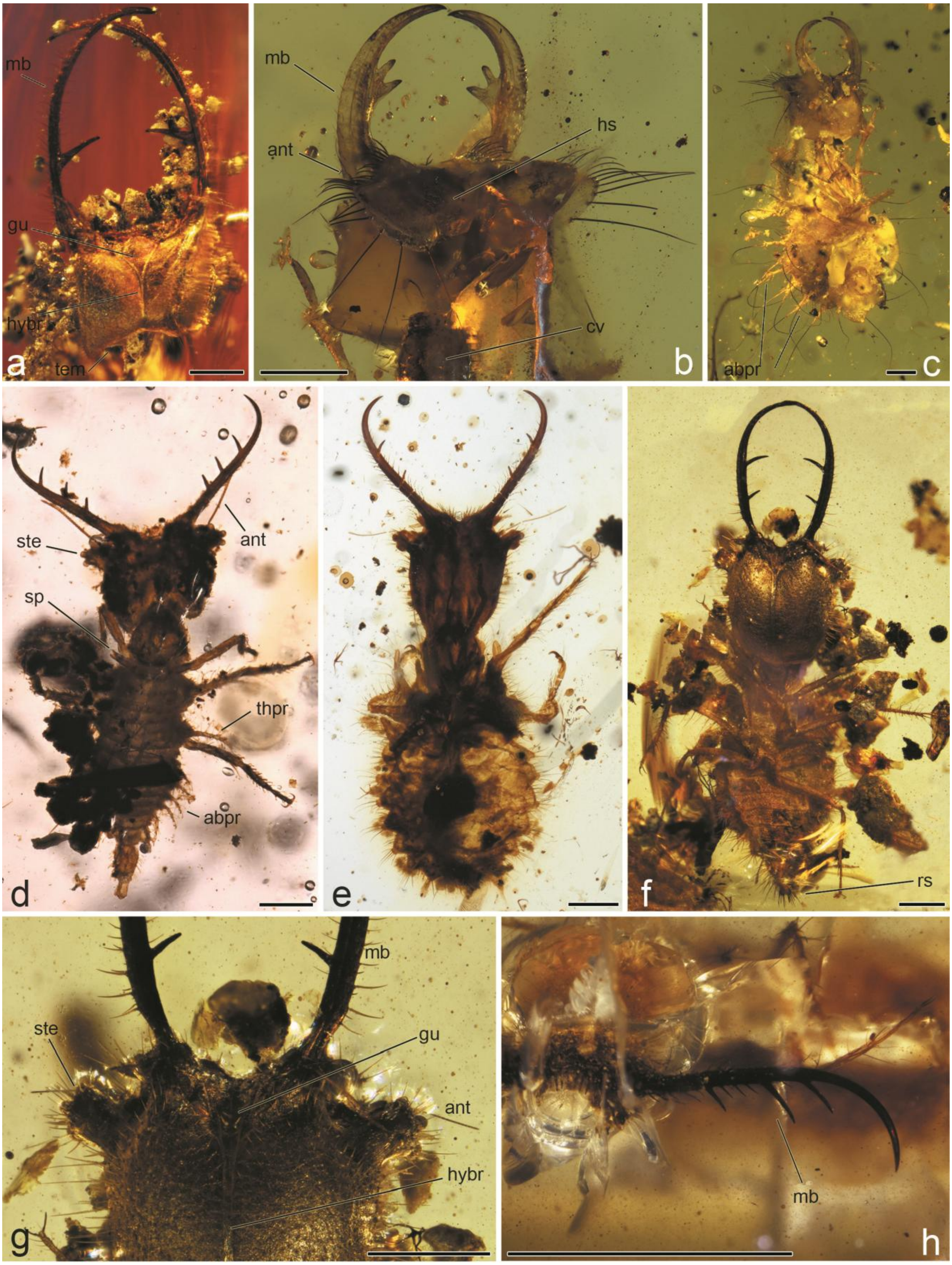


**Supplementary Figure 2.** Diversity of the larvae of Myrmeleontiformia in mid-Cretaceous Burmese amber. **a**, **b**, *Nymphavus progenitor* gen. et sp. nov., **a** holotype AMNH JCZ-Bu30, habitus, dorsal view; **b** paratype AMNH JCZ-Bu33A, head, ventral view. **c** *Burmitus tubulifer* gen. et sp. nov., holotype BA12013, dorsal view. **d** *Mesoptynx unguiculatus* gen. et sp. nov., holotype NIGP164043, habitus, dorsal view. **e**, **g** *Electrocaptivus cheni* gen. et sp. nov., holotype BA170101, **e** habitus ventral view; **g** head dorsal view. **f**, **h** *Adelpholeon lithophorus* gen. et sp. nov., holotype AMNH JCZ-Bu31, **f** habitus ventral view; **h** head ventral view. Scale bars: 0.5 mm.

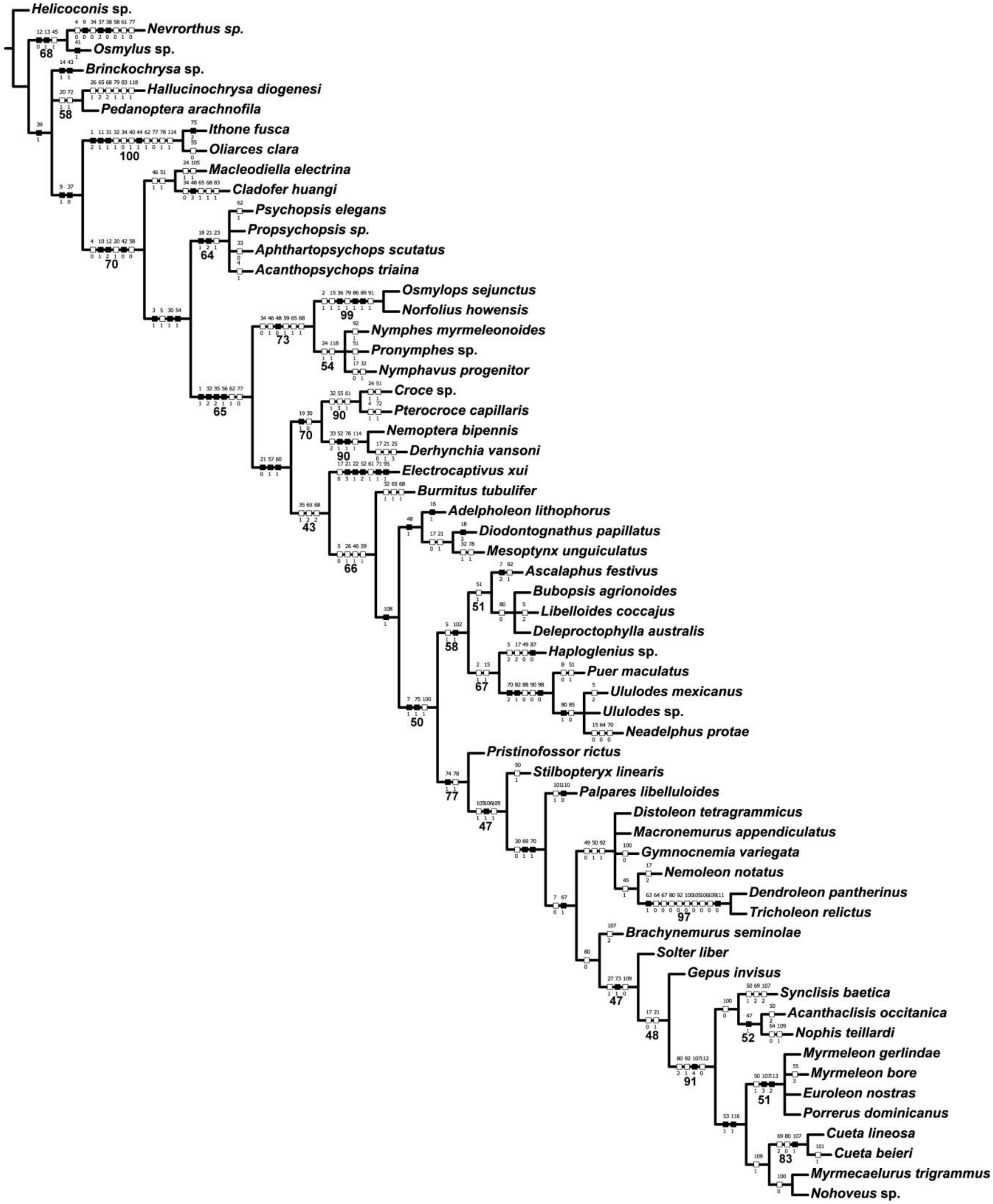


**Supplementary Figure 3.** Strict consensus tree obtained under implied weights (k=9.21875) with unambiguous character changes mapped on branches. Numbers below branches indicate maximum parsimony jackknives resampling percentiles (cut 40%).

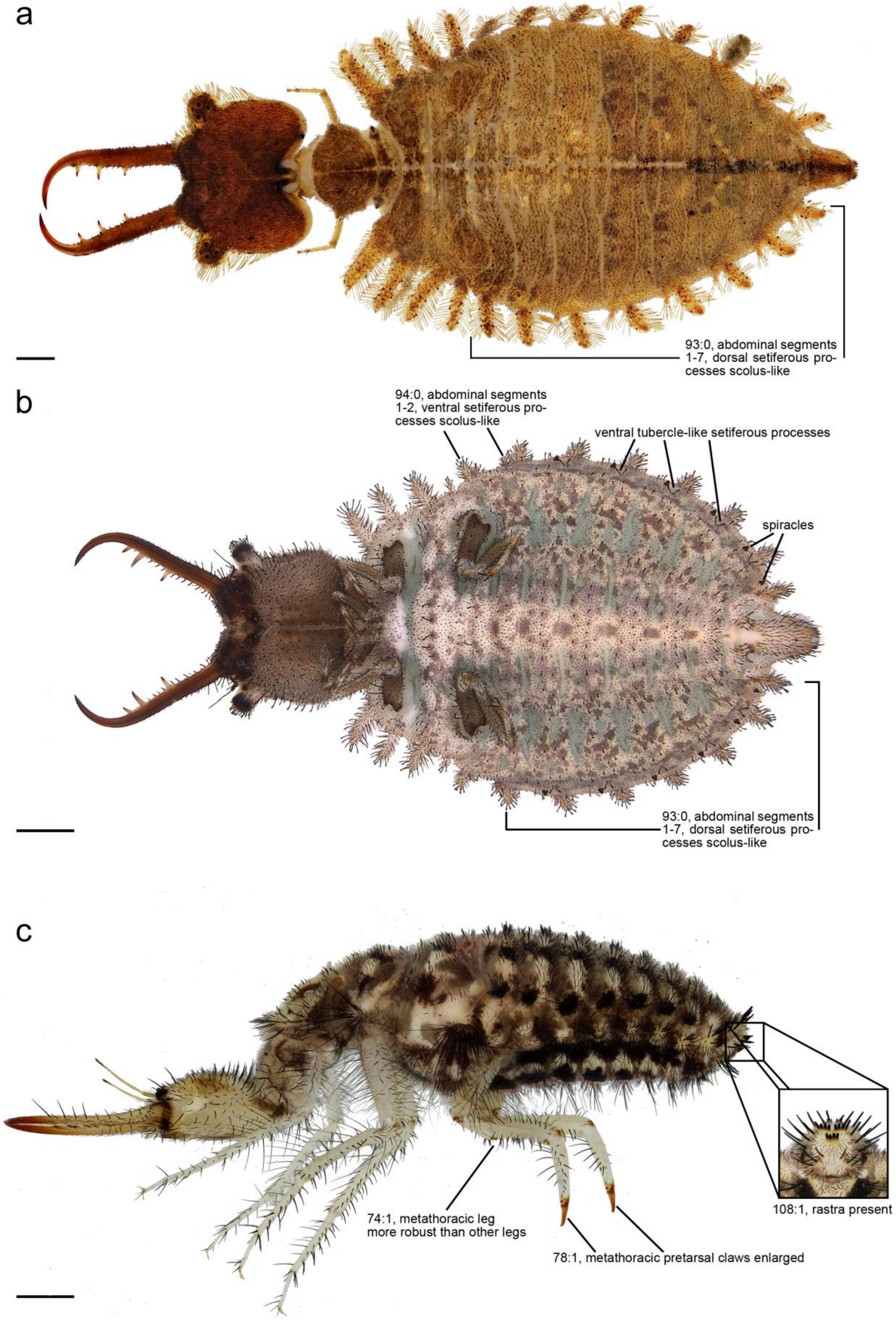


**Supplementary Figure 4.** Morphological characters used in the correlation analyses. **a** *Haploglenius* sp., Ascalaphidae, dorsal view. **b** *Libelloides ictericus* (Charpentier), Ascalaphidae, ventral view. **c** *Myrmeleon caliginosus* (Hölzel & Ohm), Myrmeleontidae, lateral view.

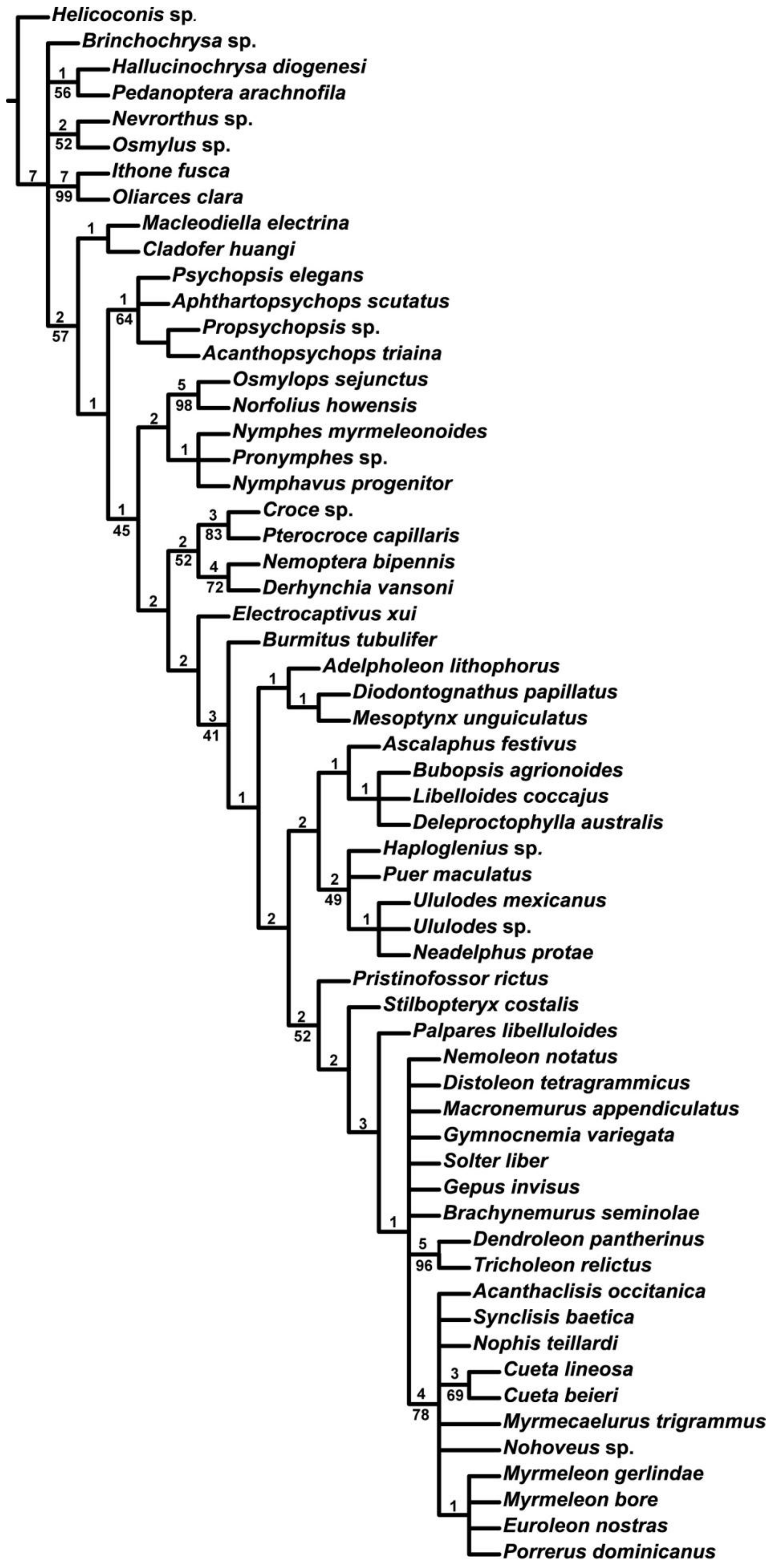


**Supplementary Figure 5.** Strict consensus cladogram of 680 trees obtained under equal weights. Numbers above branches indicate Bremer supports, while those below branches are maximum parsimony jackknives resampling percentiles (cut 40%).

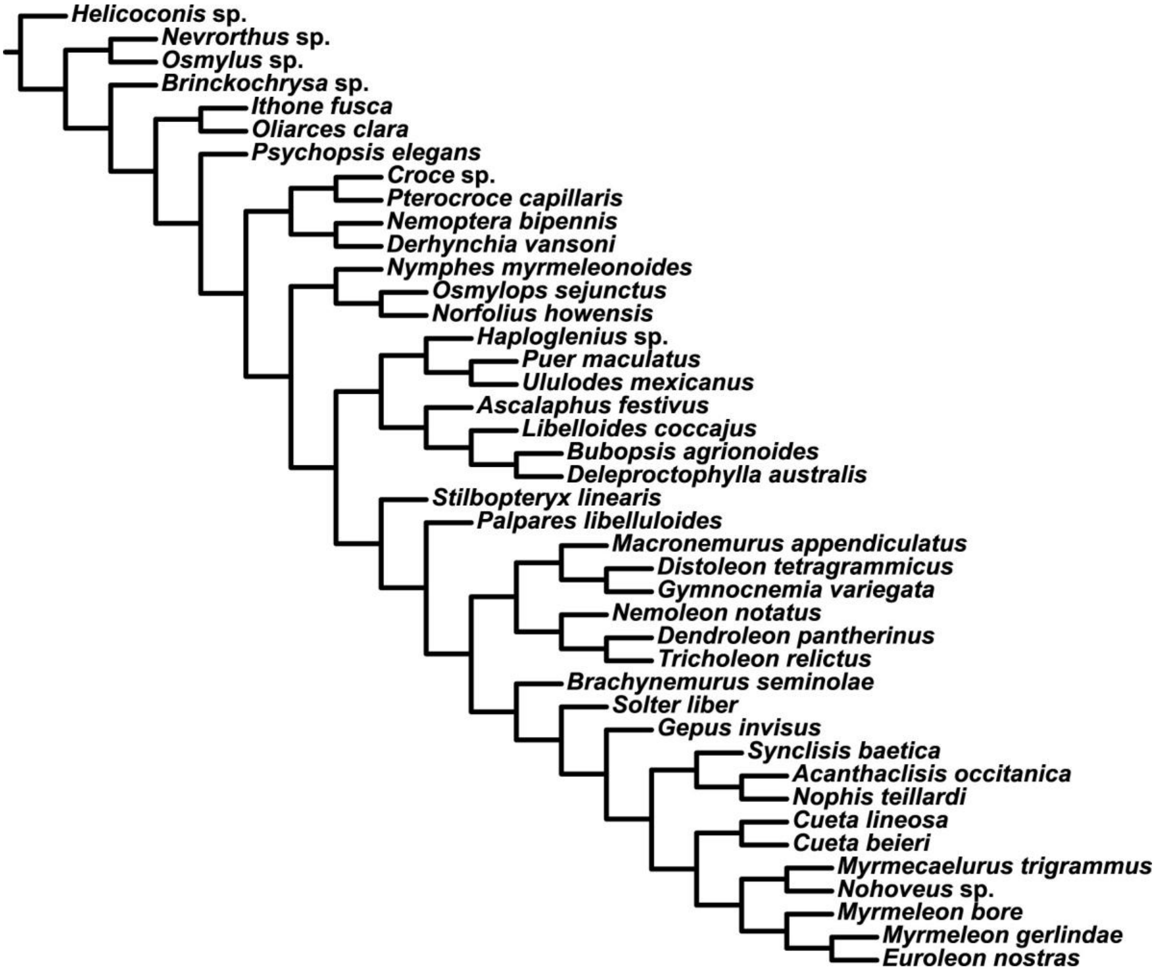


**Supplementary Figure 6.** Most parsimonious tree obtained under implied weights (k=9.21875) when fossil taxa are removed from the matrix.

**Supplementary References**

1. Engel, M. S., Winterton, S. L. & Breitkreuz, L. C. V. Phylogeny and Evolution of Neuropterida: Where Have Wings of Lace Taken Us? *Ann. Rev. Entomol*. **63**, 531–551 (2018).
