# Supplementary material for: Diverse Cretaceous larvae reveal the evolutionary and behavioural history of antlions and lacewings
Source: Nat Commun. 2018 Aug 22;9:3257. doi: 10.1038/s41467-018-05484-y (PMC6105666; doi:10.1038/s41467-018-05484-y)
Supplement: Supplementary file 3 — Description of Additional Supplementary Files [file 41467_2018_5484_MOESM3_ESM.pdf]

Description of Additional Supplementary Files

File Name: Supplementary Data 1
Description: Character matrix.
